# Long-term tactical–technical development in elite youth basketball: Brazilian national team coaches' perspectives within a Constraint-Led Approach framework

**DOI:** 10.3389/fspor.2026.1830277

**Published:** 2026-06-24

**Authors:** Jhonatan Vinícius Cintra dos Santos, Larissa Rafaela Galatti, Yura Yuka Sato dos Santos, Carine Collet, Sergio Lara-Bercial, Sergio José Ibáñez

**Affiliations:** 1 Laboratory of Studies in Sport Pedagogy, Faculty of Applied Sciences, University of Campinas, Limeira, Brazil; 2 Social Service of Industry (SESI-SP), Franca/Araraquara, Brazil; 3 Pedagogical Study Group in Sports, School of Physical Education, Physiotherapy and Dance, Federal University of Rio Grande do Sul, Porto Alegre, Brazil; 4 Carnegie School of Sport, Headingley Campus, Leeds Beckett University, Leeds, United Kingdom; 5 Training Optimization and Sports Performance Research Group, Faculty of Sports Sciences, University of Extremadura, Cáceres, Spain

**Keywords:** coaches, Constraint-Led Approach, sport development, tactical-technical content, technical content, long term athlete development, team games

## Abstract

**Introduction:**

Coaches play a central role in organizing and systematizing tactical-technical content throughout the long-term athlete development process. The present study aimed to analyze and systematize the perspectives of coaches with experience in Brazilian male youth national teams (U15 to U19), regarding priority tactical-technical content during the specialization stage, interpreting these perspectives through the lens of the Constraint-Led Approach (CLA).

**Methods:**

Semi-structured interviews were conducted with four coaches and analyzed using thematic content analysis with a hybrid (deductive-inductive) approach, organized according to the phases of the game (offense, transition, and defense).

**Results:**

The results reveal a structured progression of content across age categories, with increasing emphasis on game understanding, recognition of advantages and disadvantages, and decision-making in dynamic contexts. The concept of transforming small advantages into decisive advantages emerged as a central organizing principle in both offensive and defensive development. However, the findings also indicate the persistence of a traditional hierarchical view of technique as a prerequisite stage prior to tactical-technical development, highlighting tensions between analytical teaching models and ecological-dynamics perspectives. Furthermore, international competitive experience was identified as a relevant factor for improving adaptive decision making under pressure.

**Discussion:**

These findings reinforce the need for greater alignment between prioritized content and pedagogical methodologies, highlighting the Constraint-Led Approach as a promising pathway for tactical-technical development in elite youth contexts.

## 1 Introduction

The development of basketball players is a long-term and non-linear process influenced by multiple factors, including the organization, distribution, and progression of tactical-technical content throughout different stages of athlete development (1–4). Within this process, pedagogical decisions from the coaches play a central role, as the selection and systematization of content may be decisive for long-term athlete development (5–8).

Basketball coaches must possess deep knowledge of the sport, motivation, and empathy, integrating these competencies with the organization of training content and the selection of appropriate training methodologies (9–11). This is crucial for promoting basketball athletes development, which is related to the holistic and long-term improvement of tactical-technical skills, decision-making, psychological condition, physical and motor improvement and social adjustments (3, 7, 12, 13). Considering the basketball complexity, coaches must be able to adopt and adapt models that promote meaningful and integrated game learning (8, 14–16).

In the pedagogical scholar field, a progressive transition has been observed from models centered on decontextualized technical repetition to approaches that emphasize game understanding, decision-making, and adaptation to situational demands (17–19). Modern frameworks like the Tactical Games Approach (TGA) and Game Sense advocate for the integrated development of perception, decision-making, and execution (18, 19). Among these perspectives, in basketball, this transition is well exemplified by the Constraint-Led Approach (CLA), grounded in ecological dynamics. Rather than rehearsing an isolated jump shot or, the CLA ensures that technical skills emerge as functional solutions to specific game problems by the maintenance of perception-action coupling in representative tasks (20–22). Within this framework, coaches manipulate constraints to channel the exploration of solutions, promoting representative learning and real-time decision-making.

For example, in basketball, instead of just “knowing” a pick-and-roll strategy, the CLA fosters the player to physically adapt their dribble height or stride length based on the specific reach of a defender. It trains adaptability through constraints; by manipulating a Task Constraint (e.g., “you can only score after a change of pace”), the athlete learns to pick up affordances (opportunities for action) provided by the defender's hip position (among other game tips related to the space, opponents and teammates). The skill is never decoupled from the game's visual cues. So, the CLA promotes implicit learning by changing the environment by the coach, who stimulates tactical-technical development guided by representative tasks, more connected with the chaotic characteristic form basketball game, both in practice as in competitions (3, 23, 24).

The CLA stimulates the recognition of action opportunities (affordances) and the development of adaptive behaviors, fostering the identification and exploitation of advantageous situations (numerical, spatial, or physical) and the effective selection of actions within the game context (25–27). In this sense, tactical-technical content is understood as contextualized actions integrating perception, decision-making, and motor execution according to the demands of the game, in contrast to learning centered exclusively on isolated technical gestures (20, 21). From this perspective, discussing “what to teach” also requires considering “how to teach”, that is, how content is operationalized in representative tasks that support decision-making under realistic game constraints (22, 27).

This paradigm shift is also reflected in international athlete development frameworks. Recent studies indicate that countries highly ranked by the International Basketball Federation (FIBA) structure their development programs based on the progression of tactical-technical content, starting with contextualized individual fundamentals and gradually expanding toward collective actions throughout the development pathway (2); guidelines from Argentina, Australia, Brazil, Canada, England, United States, Spain and Australia emphasize game-centered approaches as the philosophical foundation of long-term development.

Despite these guidelines, it remains unclear how national team coaches translate such recommendations into concrete priorities for tactical-technical content during the specialization stage (U15–U19) (28), considering the different phases of the game (offense, transition, and defense). In particular, it is necessary to understand whether these professionals' recommendations converge with CLA assumptions by prioritizing decision-making, recognition of advantages and disadvantages, and functional versatility within context (21, 25). Considering that coaches are responsible for defining objectives, selecting content, and applying methodological strategies within the development process (7, 29), investigating their perspectives becomes essential for analyzing the coherence between theoretical guidelines and pedagogical practice at the elite youth level.

Within this context, the concept of Representative Learning Design (RLD) becomes central, as it proposes that training tasks should preserve the perceptual–decision-making information present in competition, ensuring functional correspondence between training and competition environments (25, 30). Task representativeness enhances the perception of affordances and behavioral adaptation, allowing tactical-technical learning to occur under ecologically valid conditions. Thus, rather than simply repeating technical gestures, the pedagogical challenge lies in structuring training environments that maintain the informational dynamics of the formal game.

Given this context, the aim of the present study was to analyze and systematize the opinions of Brazilian coaches with experience in male youth national teams regarding the priority tactical-technical content during the specialization stage (U15 to U19), interpreting these perspectives through the principles of the Constraint-Led Approach and discussing convergences and tensions with contemporary recommendations for game-based learning (21, 27).

## 2 Materials and methods

According to the research design typology proposed by Ibáñez and Feu (31) for Sport Sciences, this study is characterized as a qualitative empirical study, focused on understanding professional rationalities through the discourse of experts.

All the procedures were approved by the Research Ethics Committee of the authors' home institution. The participants signed an informed consent form, and anonymity, confidentiality, and the exclusive use of the information for scientific purposes were ensured. (Data suppressed to ensure anonymity during the submission process).

### 2.1 Participants

A purposeful sampling strategy based on expertise was adopted, selecting coaches with direct experience in Brazilian male youth national teams (U15–U19) between 2016 and 2023. The inclusion criteria were: (a) having served as head coach in national youth teams; (b) have Brazilian nationality; and (c) voluntarily agree to participate in the study.

Among the eight eligible coaches, four agreed to participate (three male coaches and one female coach). The female coach perceptions were very aligned with the male coaches' discourse, so data was analyzed with no distinction between genders. In qualitative studies centered on professional expertise, sample size is justified by informational depth and contextual specificity rather than statistical representativeness (32). The participants had coached in official competitions organized by FIBA, including South American Championships, the FIBA AmeriCup, and World Championships. Despite the rise of performance analytics, athlete recruitment and development is still dominated by the coaches' beliefs, so, there is a clear need to investigate these professional perceptions to bridge the gap between intuitive “expert eye” observations and formal tactical-technical skill development (7, 13, 33).

### 2.2 Data collection procedures

Individual semi-structured interviews were conducted, as this method is appropriate for in-depth exploration of professional perceptions, pedagogical criteria, and decision-making processes (34). The interview guide was structured around two main axes: (1) Priority tactical-technical content for the U15/16, U17/18, and U19 categories, considering offense, transition, and defense; and (2) Relevant aspects regarding the development of Brazilian youth players in comparison with international contexts**.**

The interviews were conducted online through the Google Meet® platform, whose use in qualitative research is methodologically supported in the literature (35). The average duration was approximately 75 min.

### 2.3 Data analysis

Data analysis was conducted using thematic content analysis with a hybrid (deductive-inductive) approach (34). In the first stage, deductive categories related to the phases of the game were established (offense, offensive transition, defensive transition, and defense). Subsequently, open coding was performed to identify emergent subcategories inductively, referring to technical and tactical-technical contents. This approach allows the integration of prior conceptual frameworks with the situated experience of participants, promoting contextualized and theoretically grounded analysis (34). The distinction between technical content (isolated motor execution) and tactical-technical content (contextualized action mediated by decision-making) was grounded in references from Sport Pedagogy and studies on the teaching of team sports (36, 37).

The unit of analysis consisted of the verbal statements of the interviewed coaches, which were later interpreted in light of the principles of Ecological Dynamics and the CLA, although these theoretical frameworks were not imposed during the data collection phase.

The analytical process included the following steps (34): (i) exhaustive reading and familiarization with the data; (ii) initial coding using ATLAS.ti 24 software; (iii) grouping meaning units into categories and subcategories; (iv) iterative review of conceptual coherence and consolidation of interpretations.

Scientific rigor was ensured through the criteria of credibility, internal coherence, and researcher triangulation (32). Initial coding was conducted by the first author and subsequently reviewed by two PhD researchers with expertise in sport development and qualitative research, establishing interpretative consensus.

In addition, a reflexivity process was undertaken, considering the principal researcher's previous experience as a coach in order to minimize potential interpretative biases and strengthen analytical consistency (38). The authors engaged in ongoing and in-depth discussions regarding the data and the tension between the analytical teaching models prevalent among coaches and the ecological-dynamic perspectives suggested by the literature. The composition of the authorship was decisive in this process: In addition to the first author, three other co-authors combined research activities with their work as coaches. The remaining two contributors work exclusively as researchers. This team, with its diverse roles and connections between the practical and theoretical fields, in conjunction with the literature, underpins the discussions that make up this article.

## 3 Results

The results are presented according to the phases of the game—offense, offensive and defensive transition, and defense—organized by age categories (U15/16, U17/18, and U19) and the contents classified as technical and/or tactical-technical content. The presentation follows the systematization shown in Tables 1, 2, 3, and is complemented by representative excerpts from the interviews.

**Table 1:** Offense contents suggested by the coaches for the under-15 to under-19 categories.

Contents	U15/16	U17/18	U19
Technical contents	Passes Reception Triple threat Dribbling without looking Start Diversifying Finishes Knowing how to shoot (technique/mold) Introduce the technique of pick’n roll and off screens Introduce offensive rebound techniques	Enhance content from previous ages Combine the execution of the technique with physical capabilities (intensity) Increase the repertoire of shooting	Master the basic offensive technicalities of the game
Tactical-technical content	Develop reading the opposing defense Developing the concept of advantage Develop pass type choices Develop offensive rotations after cutting/penetrating Develop the concept of a “the game without a ball”—spacing Technical versatility to play different positions Develop 1 × 1 finishing/shooting skills Simple class actions to create advantage Introduce the concept of pick’n roll and off screens Introduce the Zone Offensive	Enhance content from previous ages Deepen offensive understanding Understanding of Maintaining Advantage Improve the reading of opposing defense and where the help is coming from Offense against the weakest opponent's traits Not forcing the ball Enhance the concept of pick’n roll and off screens Develop the ability to shoot on the move and in 1 × 1 Play in different positions Develop the offense against zone through concepts	Master previous content Mastering the concept of advantage Master the tactical understanding of the game in relation to the opposing defense Master tactical endgame understanding (score/time) Ability to perform more complex actions (actions on actions/plays) Ability to create your own shot in 1 × 1

Legend: 1 × 1 - situation of one player against another.

Source: The authors.

**Table 2:** Offensive and defensive transition contents suggested by the coaches for the U15 to U19 categories.

Contents	U15/16	U17/18	U19
Defensive and offensive technical content	Develop the same offensive and defensive technical aspects	Improve the technical aspects of the previous age Mastering the long pass	Master the previous technical aspects
Offensive tactical-technical content	Tactical pre-determination during the shoot trajectory Players can perform different roles (positions) Develop the concept of fastbreak and primary transition Develop the concept of occupying space Developing the concept from small to big advantage	Use less dribbling Tactical pre-determination—players can play multiple roles Understand the concept of small to big advantage Understanding defensive adjustments (are there any individual advantages?) Develop the concept of secondary transition (actions in sequence)	Tactical pre-determination—players with specific roles Mastering the concept from small to big advantage Mastering the understanding of the opponent's defensive fit (is there an advantage over the opponent's characteristics?) Mastering the concept of space Secondary transition—more complex actions
Defensive tactical-technical content	Tactical predetermination during the shoot trajectory Run to the defense Protect the basket Stop the ball Develop an understanding of my opponent's offensive advantages	Tactical predetermination during the shoot trajectory—more specific roles Mastering the understanding of the opponent's characteristic	Tactical predetermination during the shoot trajectory—specific roles Offensive rebound Strategies to delay the opponent's transition Defend according to the opponent's characteristics

Source: the authors.

**Table 3:** Defense contents suggested by the coaches for the U15 to U19 categories.

Contents	U15/16	U17/18	U19
Technical contents	Defensive stance Footwork Close out (technique) lateral displacement BoxOut (Technique)	Improve the previous technical aspects and enhance with physical capabilities (intensity)	Mastering the technical aspects with physical capabilities (intensity and efficiency)
Tactical-technical content	Develop 1 × 1 defense Defensive positioning when my player has the ball Defensive positioning in relation to the ball (when my player is without the ball) Develop individual defense Develop the concept of defensive advantage and disadvantage Develop the understanding of the strong and weak sides of the defense What to do when my player dribbles Develop collective actions of defense off the ball—primary helping Begin the pick’n roll defense Rotations of defenders after the pick’n roll situation Begin the off-ball screens defense (follow or switch) Begin the zone defense development	Improve individual defense - half court and full court Improve understanding of defensive advantage and disadvantage Develop an understanding of the opponent's characteristics Contain the 1 × 1 Know how to position yourself in relation to the ball Improving decision-making—help or not? (overhelping) Improve the collective defense actions—primary and secondary helping Develop all pick’n roll defenses (switch, step, ice, trap, etc) Develop defending pressures—individual and zone Introduce more complex defenses—mixed	Mastering the defensive understanding of the opponent's characteristics Master the understanding of the types of defense and the why of each defense Mastering help and rotations Defensive tactical ability to change defense quickly Mastering individual defense—half court and full court Master more complex defenses

Source: the authors.

### 3.1 Offense

Table 1 summarizes the offensive technical and tactical-technical contents indicated by the coaches as priorities in the specialization phase (U15 to U19).

#### 3.1.1 Offensive technical content

In the U15/16 categories, coaches emphasize mastery of fundamental offensive skills, particularly shooting, which is understood as a structural requirement for performance at higher levels. The focus lies on technical consolidation as a foundation for future progression. Coach 3 stated:

“The most, most, most important thing is teaching the kid how to shoot. Today I work at a higher level, with the national team. If you don't have a shot, it's hard to play at a high level. And we are talking about high performance here.”

In the transition to the U17/18 category, Coach 3 further adds there is a need to expand the technical repertoire, incorporating shooting in movement, coming off screens, and finishing after lateral displacement.

“And then at the second age group, 17/18 years old, some shooting in movement. So stationary dribble and jump shot, lateral displacement, coming off an off-ball screen, something along those lines.” (C3)

Additionally, technical execution must increasingly be integrated with physical intensity:

“What's important is combining fundamentals with maximum intensity at this stage (U17/18). The physical component is combined with the technical… the more physically and technically demanding the training is, the better. Technical training needs to be very intense—that's where the level increases.” (C2)

In the U19 category, the focus shifts toward consolidated mastery of offensive technical skills and their efficient execution under greater competitive complexity.

#### 3.1.2 Offensive tactical-technical content

Regarding offensive tactical-technical content, a progression centered on game understanding and the concept of advantage/disadvantage emerges.

In the U15/16 categories, coaches prioritize simple collective actions that create initial advantages, alongside the development of defensive reading and spatial occupation, as Coach 1 states:

“I would include simple actions, direct actions, and quick actions to create an advantage. A simple off-ball screen play, a simple ball screen play—simple things.” (C1)

According to Coach 2, at the same time, there is an emphasis on developing game reading rather than offensive system complexity.

“I think it's important to have a simple system, but it's more important to develop reading than to have many plays. But it's important for the player to understand what a system is and have discipline. Promoting collective play is important at the beginning.” (C2)

At the U17/18 level, emphasis shifts toward maintaining advantages, reading defensive help, and expanding the repertoire in 1 × 1 situations.

At the U19 level, coaches highlight: Mastery of the concept of advantage; Understanding of end-of-game contexts (score and time management); Ability to execute complex actions (actions upon actions); Autonomous shot creation in 1 × 1 situations.

A recurring theme in the interviews was the difficulty players face in transforming small advantages into decisive ones.

“It's about taking a small advantage and turning it into a big one. That's something I saw at the U19 World Championship that our players struggle with today. If I could bring back only one lesson from that experience, it would be that.” (C3)

Coaches also emphasized the importance of international competition experience for developing this ability. The lack of exposure to international competitions and to higher levels of competitive demand hinders the development of these players, both psychologically—regarding their ability to perform under pressure—and in terms of their understanding of the game and decision-making.

“Against Spain, the world champion team, we played well in the first half until they changed their defense on the ball screen to NEXT (when the player closest to the pick-and-roll situation provides help from the front with the intention of stopping the ball handler). We didn't know what to do because that defense required quick decision-making—moving the ball quickly, not taking an extra dribble. Our players today don't have that capacity to read the small advantage.” (C3)

### 3.2 Offensive and defensive transition

Table 2 presents the technical and tactical-technical content associated with transition phases.

Regarding the transition technical aspects, coaches highlighted the long pass as an important skill to master at the U17/18 level. However, the primary emphasis in transition phases lies on tactical-technical elements. According to the coaches, both offensive and defensive transitions should involve tactical pre-determination. In early stages, players should experience multiple roles during transition. As age categories progress, roles become more specialized.

“At U15 and U16, everyone should be able to handle the ball in transition to develop their abilities. Later, at older ages, you start specifying each player's role.” (C1)

Understanding the difference between fast breaks, primary transition (Post-fast breaks phase. When the fast break is not possible, simple combined actions are executed—such as spacing and passing, for example—with the intention of creating an advantage against a still disorganized defense), and secondary transition (At this stage, more complex combined actions are performed with the intention of finding an advantage that was not achieved in the previous phase; it often overlaps with organized offensive sets). is also considered essential for better decision-making.

“Knowing the difference between a fast break, a primary transition, and a secondary transition is important. Then the main point is spacing, court occupation, and understanding advantage.” (C1)

Primary transition typically involves simpler actions such as ball screens, hand-offs, or off-ball screens, whereas secondary transition involves more complex sequential actions.

Regarding the phase between offense and defensive transition, the players must already know their responsibilities from the moment a shot is taken.

“Something important at this age (U15/16), actually at all ages, is understanding what I call tactical pre-determination during the ball's trajectory after a shot. Once the shot goes up, the player has a predetermined role—whether to balance the defense or go for the offensive rebound.” (C2)

For defensive transition at younger ages, coaches emphasize basic principles as: Sprinting back on defense, Protecting the rim and Stopping the ball.

“In defensive transition, the first thing is to get back and be between the ball and the rim you’re defending. Protect the rim first, then stop the ball.” (C4)

In the U17/18 and U19 categories, there is greater emphasis on identifying opponent characteristics and adjusting defensive strategies accordingly.

“It's also the moment in transition to identify where the shooter is and recognize the opponent's characteristics. So, you must slow down the fast player or stay closer to a good shooter.” (C3)

Coaches also highlight that transition actions should be executed with greater speed and intensity at advanced stages. Finally, intensifying the transition phase is essential for the development of these players, as it represents an opportunity to more closely integrate physical conditioning with technical aspects.

“In transition, players must always operate at their limit. They already understand where to run; now they must do it at a higher level—combining technique and tactics with speed and intensity.” (C2)

### 3.3 Defense

Table 3 synthesizes the defensive content identified by coaches.

#### 3.3.1 Defensive technical content

Basic defensive skills—defensive stance, lateral displacement, close-out technique, and rebounding box-out—should be introduced in the U15/16 category and progressively enhanced in later stages. Coaches also emphasize the importance of defensive rebounding:

“For me, boxing out is the most important thing—knowing how to box out and recover the ball so you can start the offense.” (C4)

In the U17/18 and U19 categories, emphasis shifts toward performing these fundamentals with greater physical intensity and efficiency**.**

#### 3.3.2 Defensive tactical-technical content

In the under-15/16, the 1 × 1 defense is considered the basis of all defensive organization, along with the understanding of the positioning in relation to the ball and the opponent.

“Reading 1 × 1 defense—before the dribble, during the dribble, and after the dribble. Sustaining 1 × 1 defense must be very well developed.” (C2)

Progressively, players begin to incorporate basic defensive concepts such as: strong-side and weak-side concepts and read the player and the ball (Understanding the player's and ball's location and their characteristics):

“Players must understand strong side, weak side, and how to read the player and the ball. At 15 or 16 they already need to know how to do that.” (C4)

Primary help (The player closest to the ball positions himself in a way that allows him to help if necessary) and simple rotations also appear as basic concepts for progression of more complex content.

“I would also focus a lot on defensive rotations, that they understand defensive rotation. To be able to press the ball more, and to press the ball well you have to have good coverage” (C3).

In the U17/18 category, defensive systems against pick'n roll and off-ball screens are developed, evolving at the U19 level into more complex systems such as full-court pressure and mixed defenses.

Coaches expressed divergent views regarding the appropriate age to introduce zone defenses, reflecting different pedagogical philosophies.

“I think it's time for the athlete to learn the principles of zone defense for sure” (C2)

“In the under-17 I would already add some zone independent of the zone.” (C3)

“I think they (under-17/18) already have to know all kinds of defense. And when you take too long to teach the zone, the player arrives at 16, 17 with a lot of defensive defects and the first defect is communication. So, sometimes, the boy is there at 12, 13, worried only about his player and even when the coach teaches how to cover, it's not the same thing. So, I think that, for example, in the U14 teaching an even defense (e.g., 2 × 3 zone) and an odd defense (e.g., 2 × 3 zone) is important.” (C4).

According to the international manuals (39), there is no consensus regarding the age at which zone defense should be introduced as a matter of knowledge. However, there is a consensus that the teaching of zone defense as a developmental tool is generally oriented toward age groups from U17 onward.

## 4 Discussion

The present study aimed to analyze and systematize the perspectives of coaches with experience in Brazilian men's youth national teams regarding the priority tactical-technical contents in the specialization phase (under-15 to under-19). In this section, we highlighted the coaches’ perspectives through the principles of the Constraint-Led Approach, discussing convergences and tensions with contemporary recommendations for game-based learning.

Overall, the results reveal a structured progression of the tactical-technical contents across age categories, with an increasing emphasis on game understanding, the perception of advantages and disadvantages, and decision-making in dynamic contexts. However, the coexistence of a traditional hierarchy centered on technique alongside perspectives more closely aligned with an ecological-dynamical rationale also emerged.

**Figure 1:**
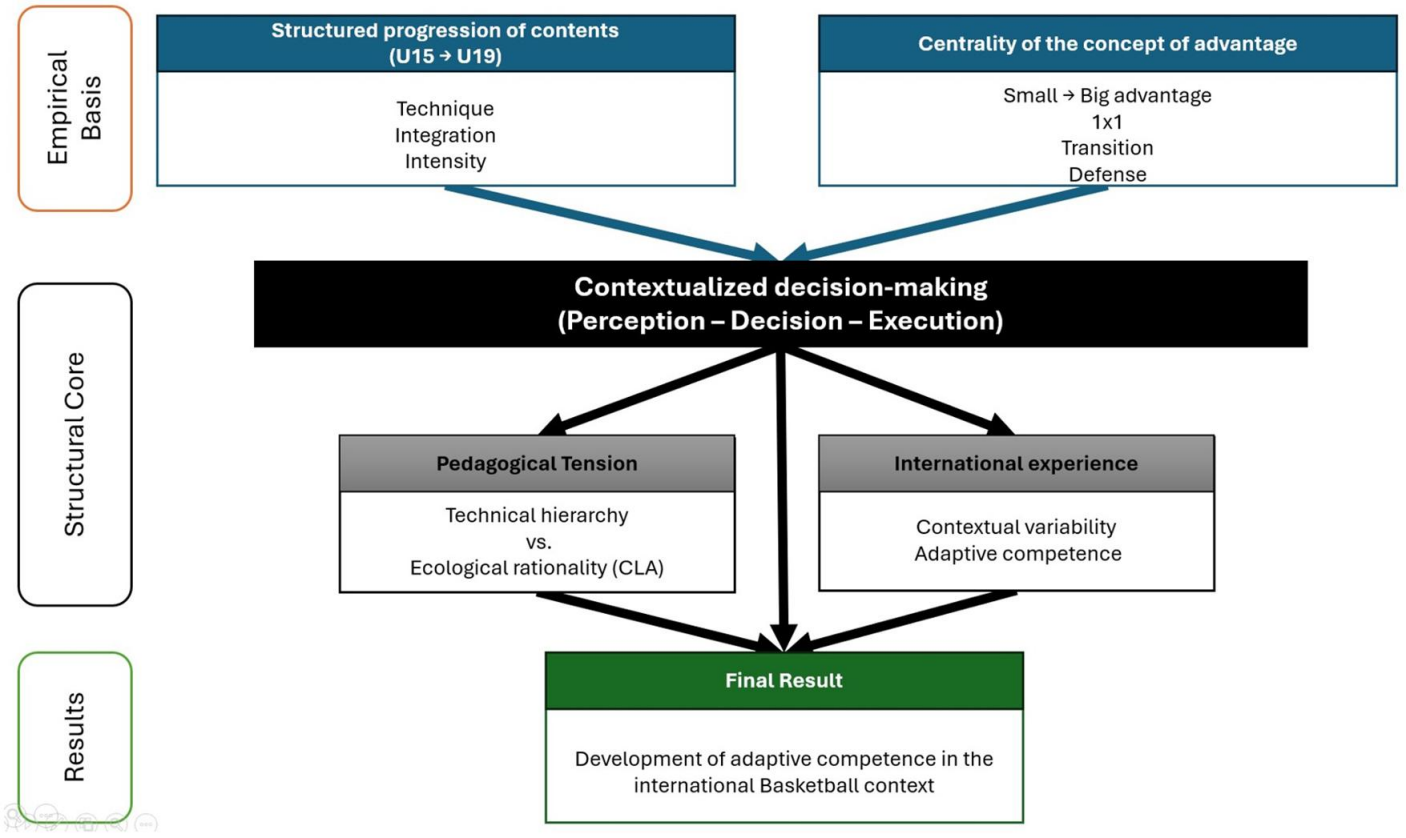
Conceptual model derived from the study findings.

Figure 1 summarizes the conceptual model derived from the study findings, organizing the progression of contents and the centrality of the concept of advantage as structuring axes of tactical-technical development. The model illustrates that these axes converge in contextualized decision-making, while simultaneously being shaped by a pedagogical tension between technique-based hierarchization and ecological rationality, and further enhanced by international experience as a mechanism for consolidating adaptive competence. Based on this conceptual framework, the discussion examines each of these components in an integrated manner.

### 4.1 Technique precedes tactics: still a traditional view of the training process

The technical and tactical-technical contents highlighted by the interviewed coaches aligns with international guidelines from countries highly ranked by FIBA (2, 3), particularly regarding the importance of developing tactical-technical contents during the specialization phase. This alignment reflects a broader and more holistic perspective on long-term athlete development.

Regarding technical contents, coaches emphasized dribbling, passing, receiving, and finishing, with particular emphasis on shooting as foundational elements for the development of more complex future contents. Although technical contents were mentioned less frequently than tactical-technical ones, it is still possible to observe a traditional view of the training process among coaches, reflecting the belief that isolated technique is necessary for learning other contents—that is, technical execution must first be mastered before learning how to play the game (36, 40).

Despite being mentioned less frequently in quantitative terms, coaches' discourse still reveals a traditional hierarchy in which isolated technique precedes tactical development. Dribbling, passing, receiving, and especially shooting are conceived as prerequisites for more complex contents. This conception sustains the idea that athletes must first learn to execute technical skills before understanding the game itself. Within this logic, emphasis is placed on *what to do* rather than *how, when, and why to do it*, that is, contextualized decision-making (36, 40). Such an approach remains widely observed in the Brazilian context, where analytical and decontextualized strategies persist in basketball instruction (14, 41), often with reduced emphasis on exploring solutions under real-game constraints (17, 42).

Despite the fact that in the last decades, scholars have rigorously critiqued the traditional “technical-first” paradigm for its linear and decontextualized nature, which often fails to prepare athletes for the chaotic unpredictability of competition (17, 43), traditional approaches remain a strong belief between the coaches. In contrast, the Constraint-Led Approach (CLA), grounded in ecological dynamics, connect the game critical elements—e.g., shot will be trained with clock pressure and/or defender's reach rather than rehearsing an isolated jump shot, the CLA ensures that technical skills emerge as functional solutions to specific game problems (21, 25).

However, recent evidence within ecological motor learning indicates that technique does not develop independently from perception and decision-making but rather emerges from the functional interaction among athlete, task, and environment (25). Therefore, maintaining a rigid hierarchy may limit the development of adaptive behaviors in complex contexts.

### 4.2 Developing tactical intelligence: centrality of the concept of advantage and decision-making

Beyond structural progression, the findings indicate that the concept of advantage/disadvantage emerges as an organizing axis of tactical-technical development. Examination of Tables 1, 2,, 3 shows that content progression increasingly emphasizes tactical-technical aspects of the game, with understanding advantage/disadvantage and recognizing opponent characteristics as fundamental elements supporting decision-making, particularly in 1 × 1 situations, both offensively and defensively.

The concept of transforming small advantages into decisive advantages emerges as a structuring core of both offensive and defensive development, especially in 1 × 1 scenarios. This perspective aligns with models that conceptualize the game as a dynamic system of continuous problem-solving, in which effectiveness depends on the ability to perceive affordances and act adaptively (44).

According to Santos Y. et al. (3), deeply understanding each athlete—including cognitive competencies related to the game—is essential, as decision-making quality depends on such knowledge. This understanding enables coaches to design training sessions with learning contents and situations that are meaningful and motivating for athletes. Within this perspective, players' capacity to make decisions in response to the unpredictability of the game is emphasized, expanding the focus from *what to do* to *how, when, and why to act* (3, 45).

This convergence between structured progression and the centrality of advantage points to a fundamental developmental core: the enhancement of contextualized decision-making. The International Basketball Federation itself recommends that game-based approaches be prioritized throughout athlete development, beginning at early stages (2), such as the Constraint-Led Approach (CLA).

One important factor highlighted by the coaches that contributes as a catalyst for the development of decision-making is the international competition. The application of training contents in formal and complex environments constitutes a central element of sport development (46, 47).

Participation in diverse competitive contexts facilitates adaptation to different tactical cultures and defensive styles (47, 48). The difficulty reported by coaches in adapting to international defensive systems may be associated with training pathways less exposed to contextual variability (39), reinforcing the need to integrate international competitive experiences into the developmental process.

This difficulty may be interpreted through the concept of adaptive expertise, defined as the ability to flexibly apply knowledge and skills in novel or unpredictable situations (49). In international basketball contexts, adaptive competence manifests in the ability to rapidly recognize new defensive configurations, adjust decisions in real time, and exploit emerging affordances under competitive pressure (50). Thus, exposure to varied contexts not only broadens competitive experience but also functions as a structural mechanism for developing adaptive expertise—an essential component of performance at international levels.

### 4.3 Technicism or game-based approach? Pedagogical tension and duality

Distinct positions emerge regarding training approaches. Part of the discourse aligns with traditional technique-centered models, particularly in offensive contents, whereas other perspectives emphasize game reading, advantage recognition, and situational decision-making, aligning with contemporary frameworks such as the CLA (21, 37). This duality reveals an epistemological tension between analytical and ecological-dynamical conceptions of basketball teaching.

The CLA has gained prominence in long-term player development by recognizing that sporting behavior emerges from interactions among player, task, and environment (21, 22). Within this framework, manipulating constraints promotes experimentation, creativity, and functional adaptation. Beyond selecting training contents, defining how those contents are operationalized becomes fundamental. Developing competent players requires structuring representative tasks that promote real-time decision-making. However, consistent implementation of CLA demands specific pedagogical knowledge and task-design competence, which are not always consolidated in coaching practice (51).

The coexistence of traditional and ecological discourses should not be interpreted as isolated inconsistency but rather as an expression of the complexity of the coach's pedagogical practice (52). Studies involving youth soccer coaches have identified similar contradictions between declared beliefs and adopted practices (53). Professional beliefs are strongly influenced by prior experiences as athletes and informal knowledge sources (54, 55). Ultimately, alignment between *what is trained* and *how it is trained* depends directly on the quality of coach education programs. In these progressive models, the coach's role undergoes a radical shift from a “transmitter of knowledge” to an architect of learning environments. Within a CLA framework in basketball, the coach does not dictate the how-to-do; instead, they manipulate task constraints (such as adjusting court dimensions, shot clock pressure, or numerical advantages—e.g., 3v2 transitions) to foster players toward discovering effective tactical solutions. The focus moves from rote repetition to representative learning design, where the information-movement coupling remains intact (3, 45). Without such educational support, the transition from traditional models to ecological-dynamical approaches tends to remain partial or inconsistent (16)

This traditional technical training persistence in Brazil may be related to the model of coach education in the country: since 1998, a bachelor's degree in Physical Education has been required by law to work as a basketball coach (5, 56). The degree in Physical Education provides relevant general training but is insufficiently specialized in basketball, in addition to recurring, more traditional, content-centered teaching approaches (57). Knowledge and content specific to basketball could have been provided through the federation system, which was not the case in basketball in the past years (58).

More recently, in 2021, The Brazilian Basketball Confederation has accredited the program offered by Instituto Basquete Brasil as its official coaching school; this program is entirely based on game-centered approaches. Also, a new law (59) considers the possibility of certification based on courses offered by national confederations or federations. In this context, continuing education grounded in critical reflection becomes essential to align pedagogical intentionality with practice (38, 60). Structured coach education programs demonstrate potential to reframe traditional conceptions and promote approaches more consistent with contemporary scientific knowledge (9, 10, 61). Certainly, other coach development strategies are needed, particularly those based on reflection and engagement with new coaching models, such as mentoring and learning opportunities for coaches (5, 10, 62, 63). These initiatives may contribute to new practices in Brazilian basketball in the years to come.

### 4.4 Contributions and limitations

This study contributes to the literature by providing an empirical and qualitative systematization of tactical-technical priorities during the specialization phase of elite youth basketball, based on the perspectives of coaches with national team experience. Unlike investigations grounded exclusively in normative documents or theoretical models, the findings demonstrate how such guidelines are interpreted and operationalized within real pedagogical decision-making contexts.

Furthermore, the study explicitly identifies a structural tension between traditional technique-based hierarchization and an ecological-dynamical rationale centered on game understanding, contributing to contemporary debates on pedagogical coherence in long-term athlete development. The centrality of the advantage/disadvantage concept as an organizing axis of developmental contents, together with the recognition of international experience as a catalyst for adaptive competence, expands understanding of the factors supporting youth performance in international contexts, offering a conceptual basis for future research and for improving coach education programs.

Despite these contributions, some limitations must be considered. First, the reduced number of participants—although justified by the qualitative nature of the study and the expertise-based sampling criteria—limits the transferability of findings to other national contexts or competitive categories. The analyzed perspectives reflect the specific reality of coaches involved in Brazilian men's youth national teams and may not fully represent other developmental structures or cultural contexts. Future research could expand the sample to include coaches from different countries, competitive environments, and women's categories, enabling international comparative analyses and identification of potential structural patterns or divergences in tactical-technical development.

Second, the study relied exclusively on coaches' discourse analysis, without incorporating direct observation of training sessions. Consequently, it was not possible to verify the extent to which declared conceptions are effectively translated into pedagogical practices aligned with the stated principles. Future studies could adopt multimethod designs combining interviews, systematic training observation, and task analysis, enabling deeper understanding of the relationship between pedagogical beliefs, methodological decisions, and real teaching behavior. Such advancements would help reduce the gap between discourse and practice and strengthen the applicability of findings in coach education program development.

## 5 Practical applications

The findings of this study allow the identification of concrete implications for planning and increasing the training process in elite youth basketball. From a pedagogical standpoint, from the Constraint-Led Approach perspective, coaches should transition from decontextualized “technical-first” drills toward the design of Representative Learning Tasks that preserve the perception-action coupling essential for elite performance. Instead of practicing isolated shooting or dribbling, training sessions should be structured around the manipulation of specific task constraints—such as varying the shot clock, court dimensions, or defensive pressure—to force the emergence of functional technical solutions. For example, to develop the identified priority of “transforming small advantages into decisive ones,” coaches can implement small-sided games (e.g., 3v2 or 4 × 3 transitions) that require players to immediately perceive and exploit spatial or numerical affordances, rather than following a pre-determined offensive script.

Furthermore, these findings highlight a critical need for Coach Education Programs to address the persistent “technical-tactical hierarchy” tension by promoting a more integrated ecological-dynamics rationale. Professionals should be encouraged to consider the National Coach opinions. to reflect on their own practice, to be exposed to other approaches as the CLA to to foster critical thinking and adaptive expertise in youth players to consolidate decision-making autonomy and ensure that tactical intelligence is developed in contexts that mirror the chaotic complexity of the formal game.

## 6 Conclusion

This study systematized the perspectives of Brazilian youth national team coaches, revealing a progressive organization of developmental contents (U15–U19) aligned with contemporary international guidelines. While findings show an increasing emphasis on game intelligence and decision-making, a tension persists between traditional “technique-as-a-prerequisite” models and modern ecological-dynamical approaches. This highlights a critical need for pedagogical coherence, specifically through the Constraint-Led Approach that integrates perception and action within representative training tasks. Ultimately, fostering “adaptive expertise” in basketball requires coach education programs that bridge the gap between prioritized tactical contents and the operational consistency of contextualized, game-based training.

## Data Availability

The datasets presented in this article are not readily available because this research is based on individual interviews with basketball coaches. As part of the confidentiality agreement, raw data is not available. Requests to access the datasets should be directed to Larissa R. Galatti – lgalatti@unicamp.br.
